# Broadband single-molecule excitation spectroscopy

**DOI:** 10.1038/ncomms10411

**Published:** 2016-01-22

**Authors:** Lukasz Piatkowski, Esther Gellings, Niek F. van Hulst

**Affiliations:** 1 ICFO—Institut de Ciencies Fotoniques, The Barcelona Institute of Science and Technology, 08860 Castelldefels (Barcelona), Spain; 2 ICREA—Institució Catalana de Recerca i Estudis Avançats, 08010 Barcelona, Spain

## Abstract

Over the past 25 years, single-molecule spectroscopy has developed into a widely used tool in multiple disciplines of science. The diversity of routinely recorded emission spectra does underpin the strength of the single-molecule approach in resolving the heterogeneity and dynamics, otherwise hidden in the ensemble. In early cryogenic studies single molecules were identified by their distinct excitation spectra, yet measuring excitation spectra at room temperature remains challenging. Here we present a broadband Fourier approach that allows rapid recording of excitation spectra of individual molecules under ambient conditions and that is robust against blinking and bleaching. Applying the method we show that the excitation spectra of individual molecules exhibit an extreme distribution of solvatochromic shifts and distinct spectral shapes. Importantly, we demonstrate that the sensitivity and speed of the broadband technique is comparable to that of emission spectroscopy putting both techniques side-by-side in single-molecule spectroscopy.

## Abstract (web summary)

In single-molecule spectroscopy typically only emission spectra are recorded. Here, the authors develop a technique to record single-molecule excitation spectra under ambient conditions, which is robust against blinking and bleaching, and reveals a large distribution of spectra across the molecule ensemble.

## Graphical abstract

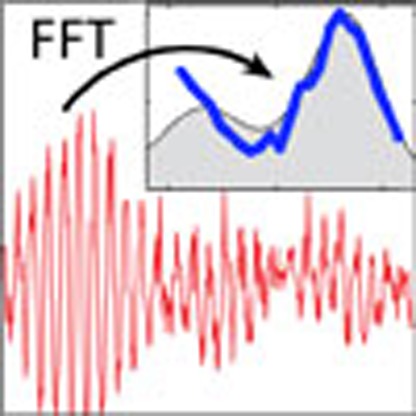

Optical spectroscopy is a primary analysis tool underlying almost any field of science; absorption, emission and excitation spectra are routinely recorded on bulk samples. The advent of single-molecule detection pushed spectroscopy to a next level: probing molecules one by one unravels inhomogeneities and dynamical processes that are otherwise hidden in the ensemble average. Intrinsic molecular diversity and distinct interactions of molecules with their nanoenvironment lead to wide distributions of their spectral properties. Pursuing a single molecule in time reveals discrete dynamics such as blinking^1^ and spectral diffusion^2^,^3^,^4^,^5^,^6^. To date single molecules, quantum dots, nanoparticles and proteins are detected and tracked with wide-ranging applications in molecular biology, polymer chemistry, nanoscopy and so on.

Historically, the first single molecules were detected through absorption^7^ and fluorescence excitation^8^ at cryogenic temperatures—conditions leading to high photostability and large absorption cross-section have allowed the recording of both excitation and emission spectra of individual molecules^5^,^6^,^9^,^10^,^11^,^12^. Far superior signal-to-noise ratios, however, are obtainable with the background-free fluorescence-based detection as opposed to absorption-based spectroscopy; as a result detection of single molecules through their fluorescence has established as the predominant method of choice.

The vast majority of applications in biology and chemistry involve the study of molecules in their natural state that is under ambient conditions. At room temperature, however, the significantly broadened absorption lines combined with a substantial reduction of the photostability and absorption cross-section demands more sensitive fluorescence detection methods and spatial separation of individual molecules. The introduction of near-field imaging^13^ and non-invasive confocal microscopy^14^,^15^ have guaranteed the necessary sensitivity and spatial resolution, however, the limited number of photons emitted before photobleaching still puts a major constraint on all room temperature single-molecule spectroscopy techniques. Therefore, only time and detection efficient spectroscopic schemes are feasible, limiting room temperature experiments largely to the recording of emission spectra^15^,^16^,^17^,^18^.

In recent efforts to go beyond single-molecule emission spectroscopy, individual molecules have been detected using photothermal contrast^19^, scattered light^20^,^21^, plasmonic structures^22^ and even direct detection of a single molecule through absorption has been demonstrated at room temperature^23^,^24^,^25^. Although very interesting, these approaches are far from routine and their main drawback is that they typically lack spectral information.

Previously, Stopel *et al*.^26^ determined the Stokes shift of individual molecules recording both excitation and emission spectrum at room temperature. Against the general believe, they found that the Stokes shift varies between individual molecules. In this study, the excitation spectra were recorded by serial scanning of the narrowband excitation wavelength derived from a white-light continuum. A disadvantage of this approach, however, is that blinking and bleaching of the molecule compete with the sequential scanning, which may result in an incomplete excitation spectrum. Recently Weigel *et al*.^27^ were the first to close a coherent control loop on a single molecule, showing that essentially the excitation spectrum is probed.

The ability to routinely perform single-molecule excitation spectroscopy under ambient conditions side-by-side to the already well-established single-molecule emission spectroscopy would be highly valuable. The crucial difference between the two spectroscopies is that emission spectra probe the final spontaneous, nanosecond decay to the ground state and its vibrational progression, while excitation spectra explore the excited state, its vibrational manifold and any intermediate short-lived (ps) states towards the decay. The excitation spectra are particularly sensitive to coupling between molecules or the nanoenvironment of the single molecules in general. Having both spectra at our disposal, a complete picture of the spectral characteristics and excited state dynamics of the molecule emerges.

Beyond the fundamental properties of individual molecules, such as assymetry in absorption–emission spectra, origins of blinking–bleaching dynamics and intra/intermolecular inter-system-crossings, excitation spectra provide valuable information in applied fields such as environmental sciences, medicine and material sciences, just to name a few. For example excitation spectra are used to determine the nature, relative quantities and composition of chromophores in plants, photosynthetic units and coral reef matter^28^,^29^. Even in the studies on photodynamic therapy for cancer treatment, excitation spectra are used to non-invasively determine *in vivo* the penetration depth of drugs in skin^30^. However, in such applications the multichromophoric composition leads to complex photophysics including energy transfer, self-quenching, reabsorption and reemission, complicating the multicomponent analysis of excitation spectra. Clearly the ability to measure excitation spectra of molecules in extremely small volumes and at interfaces is desirable. A novel approach to single-molecule excitation spectroscopy at ambient conditions would provide sufficient sensitivity to probe even monolayers of such samples with diffraction-limited spatial resolution.

Here we demonstrate a fast and efficient broadband excitation spectroscopy method to record single-molecule excitation spectra at room temperature. Our results uncover heterogeneities in the excitation spectra with unexpectedly large spectral shifts over 100 nm between individual molecules, which have remained beyond observation so far. Exploiting an ultra-broadband laser, we probe the entire excitation spectrum at once while recording the resulting fluorescence response. Different excitation wavelengths are sampled when scanning a time delay between two interfering broadband (over 100 nm) laser pulses. The time-dependent fluorescence response then yields the excitation spectrum through a Fourier transformation. Conceptually, our method is a pulsed single-molecule approach, in contrast to earlier continuous-wave Fourier excitation spectroscopy on bulk samples^31^,^32^. An important advantage of interferometric excitation over scanned narrowband excitation is that in the presence of blinking and even bleaching (provided that the main part of the interferogram around Δ*t*=0 is largely intact) full spectral information is still contained in the measured (though incomplete) interferogram, with no significant effect on the quality of the measured excitation spectrum. The insensitivity of the interferometric approach to fluorescence intensity fluctuations and spectral jumps has also been shown for interferometric detection of the emission spectra of individual molecules at cryogenic temperatures^33^. Moreover, the coherence and fs-resolution of the interfering broadband pulse-pair can be exploited to control the excited state of individual molecules before fluorescence emission^34^.

## Results

### Inhomogeneity of single molecules

**Figure 1:**
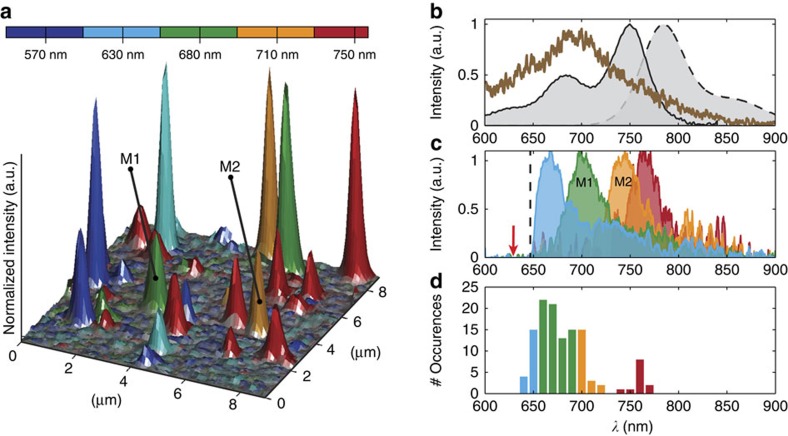
Heterogeneity of single molecule emission. (**a**) A composite confocal image of dispersed QDI molecules in a PMMA matrix constructed from a series of five normalized images recorded at different excitation wavelengths. The assigned colour corresponds to appearance of the molecules in the image for a specific excitation wavelength. The height of the peaks reflects the relative fluorescence intensity between the molecules. (**b**) Absorption (black solid line) and emission (black dashed line) spectra of a solution of QDI in PMMA/toluene mixture. The brown solid line indicates the absorption spectrum of QDI molecules embedded in a solidified PMMA matrix. (**c**) Exemplary emission spectra associated with molecules absorbing at different wavelengths. Molecules M1 and M2 correspond to the two molecules from the composite confocal image in **a**. The red arrow indicates the excitation wavelength (633 nm), the black dashed line indicates the cutoff wavelength of the long-pass filter. (**d**) A histogram representing the positions of the maxima of the emission spectra for all measured molecules. The assigned colours correspond to the excitation wavelengths indicated in panel (**a**).

All our measurements of the excitation and emission spectra were performed on single quaterrylene diimide (QDI) molecules derived from a rylene dye family^35^,^36^. Rylene dyes have been extensively used in single-molecule experiments because of their extraordinary brightness and photostability^23^,^33^,^37^,^38^,^39^. The absorption spectrum of a solution of QDI in toluene peaks around 750 nm, as shown in Fig. 1b (solid, black line), whereas the emission spectrum of the same QDI solution (upon excitation at 633 nm) is Stokes shifted to 780 nm (black dashed line).

We first turn our attention to the emission spectra of individual QDI molecules, which exhibit intriguing properties^40^. In Fig. 1a we show a composite confocal fluorescence image of QDI molecules embedded in a polymethyl methacrylate (PMMA) matrix. The image is constructed by superimposing five individually recorded images of the same area using different excitation (detection lower limit) wavelengths: 570 nm (610 nm); 630 nm (648 nm); 680 nm (730 nm); 710 nm (740 nm); and 750 nm (778 nm). Before the composition, images were normalized to unity. The height of the peaks reflects the relative fluorescence intensity between the molecules. Each molecule was labelled with a different colour according to the spectral range, in which it starts absorbing and emitting light. Molecules excited at the longest wavelength (750 nm) are labelled red; molecules that only start emitting when excited at 710 nm are labelled orange and so on. The efficient excitation of QDI molecules at short excitation wavelengths (around and below 650 nm) is surprising, as the absorption of QDI solution in this spectral range is very low (below 20% of the maximum). We have found, however, that the QDI absorption spectrum undergoes a significant blueshift (hypsochromic shift) and modification of the spectral shape, upon embedding the molecules in a PMMA matrix (see brown spectrum in Fig. 1b and Supplementary Note 1). The QDI/PMMA absorption spectrum is broadened and lacks the vibrational progression feature, which is clearly resolved in the solution absorption spectrum. The strong spectral shifts imply that to understand the spectral properties of QDI in PMMA, it is necessary to disentangle the excitation spectra of individual molecules.

To assess the spectral variation, we measured a total of 122 single-molecule emission spectra at excitation (detection) wavelength of 633 nm (above 655 nm). The emission spectra of four typical molecules representing the light blue, green, orange and red type molecules in the composite confocal image (Fig. 1a) are shown in Fig. 1c. The main emission peak position of the emission spectra was determined with a Gaussian fit, and the distribution of peak positions is plotted in Fig. 1d.

The presented data evidently shows that the emission spectra of individual QDI molecules in the PMMA matrix exhibit a remarkably large distribution of spectral shifts towards lower wavelengths with respect to the emission spectrum of the QDI ensemble solution spectrum. Moreover, it underlines that the maximum fluorescence peak can shift by more than 100 nm compared with the ensemble solution which has a maximum peak at 780 nm (see Fig. 1b, black, dashed line). The confocal image (Fig. 1a) illustrates that a significant amount of molecules only becomes visible when changing the wavelength by more than 150 from 750 to 570 nm. Conversely, the majority of molecules, emitting upon 750 nm excitation are not visible anymore when excited at 570 nm. The absorption of individual molecules shift spectrally more than the width of the QDI solution absorption spectrum (roughly 160 nm) and, thus, will remain unnoticed when the excitation wavelength is chosen solely based on the solution spectrum. This risk of selective detection is clearly observed in Fig. 1d. Both shape and position of the detected fluorescence emission distribution depend on the choice of excitation wavelength. These findings clearly indicate that a broad range of excitation wavelength is necessary to probe the entire distribution of emission spectra. The majority of single-molecule detection methods rely on the acquisition of fluorescence upon narrowband excitation with just a single wavelength, which limits and biases the quantitative analysis of spectral distributions in single-molecule experiments.

Although quite large, the observed spectral shifts and spectral variability are not uncommon in single-molecule emission spectra. In fact, analogous effects have been reported for a number of other molecules, including perylene diimide (molecular analogue of QDI) embedded in polyvinyl alcohol (PVA), for which ∼40-nm wide distribution of emission spectra has been found because of the heterogeneity of the polymer matrix sites as well as twisted conformations of the core^41^,^42^. It has also been shown that changes in the photophysical form of the green fluorescent protein in polyvinyl alcohol lead to a spectral variations of nearly 100 nm due to π-stacking interactions, extended chromophoric π-systems and photoactivation^43^. Comparable spectral shifts have been measured in single carbocyanine dye molecules adsorbed on bare glass along with strong alterations in the shape of the vibronic bands^44^.

Clearly, the dramatic variability of the observed emission spectra should be reflected in the excitation spectra, while differences give clues on the nature of the heterogeneity. In the following, we, thus, focus on the challenge to measure the excitation spectra of individual molecules to address their large spectral variability and directly compare excitation and emission spectra.

### Broadband excitation spectroscopy

**Figure 2:**
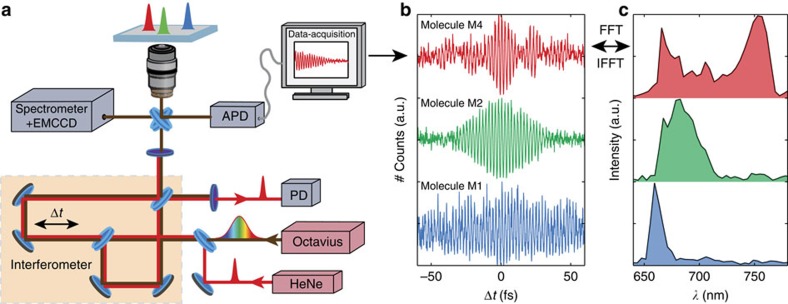
The concept of broadband single-molecule excitation spectroscopy. (**a**) Schematic representations of the experimental set-up: a broadband fs laser (Octavius) is split in two branches, and a molecule of choice excited by the pulse-pair, while scanning the time delay Δ*t*. (**b**) Three typical fluorescence interferograms of individual QDI molecules (M1, M2 and M4), each exhibiting a specific delay time-dependent fluorescence response and (**c**) their corresponding Fourier transformations. For full fluorescence interferograms see Supplementary Fig. 1. The typical contrast between the background count level and the fluorescence signal is 1/5–1/8 (see Supplementary Fig. 1). FFT, fast Fourier transform; IFFT, inverse fast Fourier transform

The experimental set-up is sketched in Fig. 2a (for details see Methods). The broadband laser excitation spectrum is derived from a Ti:sapphire oscillator and covers the wavelength range 655–770 nm, with 12 fs pulse duration. After propagating through a Mach–Zehnder interferometer including a delay line with a sub-fs interpulse delay precision, the interfering laser pulses with interpulse delay Δ*t* excite a single molecule whose fluorescence response is detected. The total fluorescence intensity *F*(*ω*) is directly proportional to both the molecular excitation probability (absorption cross-section) *F*_QDI_(*ω*) and the laser spectral power *F*_laser_(*ω*) at excitation frequency *ω*. Each spectrum *F*(*ω*) has a Fourier-related time-dependent interferogram *H*(Δ*t*). A different combination of wavelengths is sampled at each delay line position and information on the excitation spectrum (that is, absorption cross-section) is contained in Δ*t* dependent fluorescence intensity.

When exciting a molecule in its linear optical response regime, the measured fluorescence response *H*(Δ*t*) is the convolution of the laser interferometric autocorrelation function *H*_laser_(Δ*t*) and the interferogram of the excitation spectrum *H*_QDI_(Δ*t*) of the molecule: 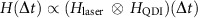. Using the convolution theorem, the Fourier transformation of *H*(Δ*t*) turns into the product of the laser spectrum and QDI excitation spectrum: 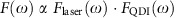. Dividing *F*(*ω*) by the laser spectrum *F*_laser_(*ω*), we directly obtain the excitation spectrum of the molecule.

In Fig. 2b we show three examples of the time-dependent fluorescence response of single QDI molecules. Differences in the extent of the interference and beating in the interferogram recordings are a clear sign that each of the molecules interacts differently with the broadband excitation laser. Molecule M4 (red) shows a beating pattern on a time scale of ∼25 fs, a clear signature of the presence of a superposition of (at least) two distinct frequency bands. The other two interferograms (molecule M1 and M2) clearly lack this feature. The corresponding Fourier transforms are shown in Fig. 2c. As expected, the red spectrum shows two bands whereas the other two (green, blue) show only one. Since the laser spectrum was identical in all three cases, the clear disparities between these spectra indicate differences in the excitation spectra of the molecules. The narrow spectra of M1 and M2 are either caused by a very narrow excitation spectrum of the molecule or the molecular excitation spectrum being shifted towards lower wavelengths, out of our laser spectral window.

### Single-molecule excitation spectra

We have measured the fluorescence response of 25 molecules along with interferograms of the excitation laser. The excellent photostability of the QDI molecules enabled the recording of several consecutive interferograms for most molecules (a total of 95 individual interferograms).

**Figure 3:**
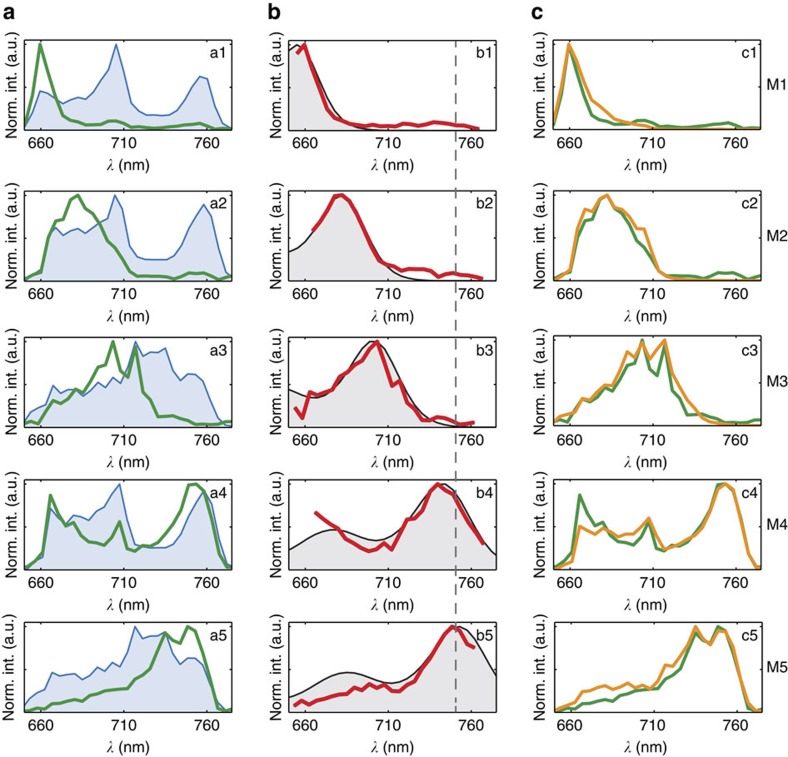
Excitation spectra of single molecules at room temperature. (**a**) Experimental spectra of single molecules (green lines) for a series of five molecules (M1:M5), together with the laser spectrum (blue, shaded). (**b**) Single-molecule excitation spectra (red lines) obtained by dividing the experimental single-molecule product spectra by the corresponding laser spectra. Shaded grey spectra in **b** represent the ensemble absorption spectra shifted accordingly for direct comparison. The dashed line indicates the position of a maximum of the QDI ensemble absorption spectrum at 750 nm. (**c**) Single-molecule product spectra (green lines) compared with reconstructed theoretical spectra (orange lines) obtained by multiplying the appropriately shifted ensemble QDI absorption spectrum (grey, shaded) by the experimental laser spectra (blue, shaded).

In Fig. 3 we show distinct excitation spectra for five single QDI molecules (M1:M5). In Fig. 3a1–a5, we present the Fourier transformations of the fluorescence response (that is the product spectrum *F*(*ω*)) measured on each individual molecule (solid line, green) and the laser spectrum measured aside the molecule (shaded area, blue). Dividing the green product spectra by the blue-laser spectra directly yields the excitation spectra of the molecules, which are presented in Fig. 3b1–b5 (solid line, red). For comparison, we also show the ensemble solution absorption spectrum (shaded area, grey), which has been spectrally offset from the ensemble position to match the measured single-molecule excitation spectra. Here we assume that only the absolute spectral position, and not the vibrational progression of individual QDI molecules undergoes significant change under encapsulation of the molecules in the PMMA matrix. The excitation spectra of another 6 individual QDI molecules (M6:M11) are shown in Supplementary Fig. 2.

We find that the measured single-molecule excitation spectra are strongly shifted compared with the ensemble absorption spectrum. Interestingly, we can, however, reproduce the experimental product spectrum by multiplying the measured laser spectrum by the appropriately spectrally shifted QDI ensemble solution spectrum. The comparison between the measured (solid line, green) and reproduced (solid line, orange) single-molecule product spectrum is shown in Fig. 3c. The two curves agree quite well for all the measured molecules.

As can be seen in Fig. 3a, we have used two different shapes of laser spectra for the measurements (M1, M2 and M4—first shape; M3 and M5—second shape). We thus confirm that our approach and its sensitivity do not depend on the spectral shape of the laser. Each of the 25 molecules we measured exhibited a different excitation spectrum. While we observed hypsochromic shifts of up to 100 nm (see M1), we did not observe any molecules red-shifted by more than a few nm (see M5) with respect to the ensemble solution spectrum. The use of broadband excitation allows us to directly sample the distribution of single-molecule spectra over a 100 nm broad band, which with narrowband excitation would be difficult to probe. Because the measured excitation spectra of the molecules with small hypsochromic shifts (that is, large overlap with our laser spectrum) indeed resemble the ensemble solution absorption spectrum, we expect that the more (above 100 nm) blue-shifted molecules exhibit similar excitation spectrum containing the vibrational progression. For M1, M2 and M3, however, the vibrational bands are outside the spectral window of our broadband excitation laser.

### Variations in the shape of the excitation spectra

**Figure 4:**
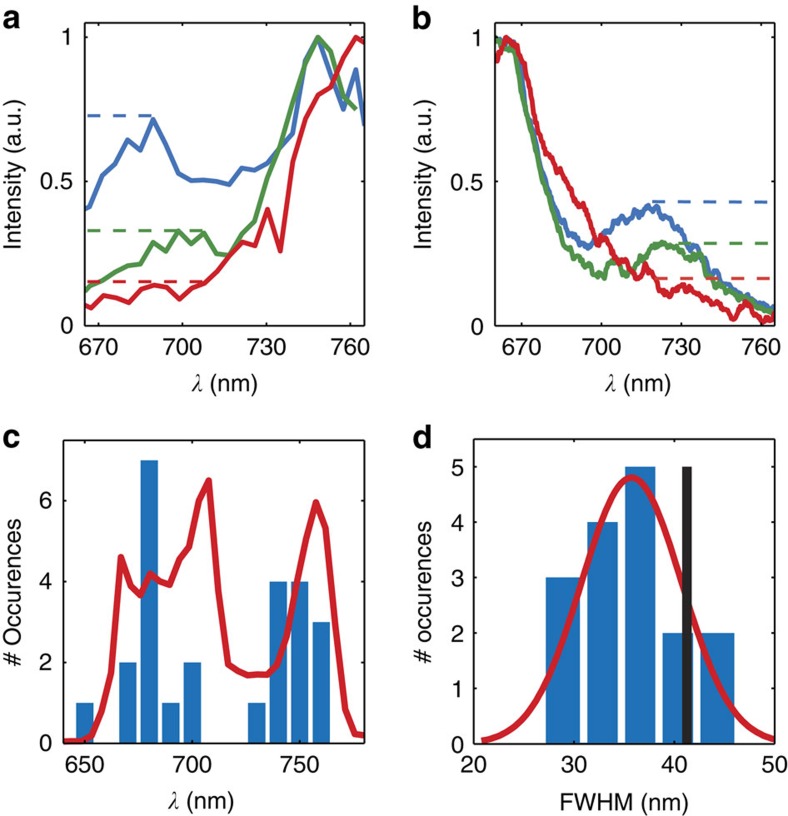
Heterogeneity of the excitation and emission spectra. Comparison of three single-molecule excitation (**a**) and emission (**b**) spectra showed with red, green and blue lines. Both types of spectra show a similar spread of relative peak intensities and inter-vibronic band distances (see corresponding colours). (**c**) A histogram presenting spread of the 0'–0'' transistion positions of all measured excitation spectra along with the laser spectrum (red). (**d**) Distribution of the 0'–0'' transition peak widths along with a fitted Gaussian spread function. The vertical black line indicates the width of the transition in the bulk solution spectrum.

Upon closer inspection, our results do display more spectral variability than just the spectral shifts shown in Fig. 3. The single-molecule excitation spectra exhibit a variety of inter-vibronic band distances, spectral band widths and relative peak intensities. In Fig. 4a, we demonstrate three typical excitation spectra for which the main electronic transition band (0′–0′′, number indicating vibrational level, single/double apostrophe noting ground/excited electronic state) overlap the best. Differences in the relative peak intensities are clearly visible. Moreover, changes in the distance between the two vibronic transitions (0′–0′′ and 0′–1′′) are noticeable in the spectra. Similar spectral variations can also be found in the emission spectra, which for comparison we show in Fig. 4b. These observations illustrate that we have achieved sufficient sensitivity in the measured excitation spectra to probe the effect of the nanoenvironment on the vibrational modes of individual molecules.

We determined a spread of the main transition positions for all the measured excitation spectra in Fig. 4c. We find that the 25 excitation spectra are rather homogeneously distributed across the laser spectrum (shown in red). As the fluorescence is detected above 785 nm, less emission is collected for molecules that are very blue-shifted compared with those absorbing at longer wavelengths, making the former underrepresented in our choice of excitation range. Furthermore, the shape of the laser spectrum may also have an indirect effect on the measured distribution—in case the laser spectrum would be more intense on the red side than on the blue side, the molecules absorbing on the blue side would be even further underrepresented as they would appear even dimmer in the confocal image. This once again pinpoints the importance of the right choice of excitation wavelength, detection range and even intensity distribution of the laser spectrum. We did not find any correlation between the position of the main electronic transition band and its spectral width. In forthcoming experiments, utilizing a broader laser excitation spectrum, our approach should allow us to study possible correlations between the spectral position of the excitation spectrum and the spectral properties of the vibrational progression (separation, intensity and widths).

For completeness, we have analysed the width of the main transition band (0′-0′′) for 16 out of 25 measured excitation spectra (those for which the main transition band was completely overlapping with the spectral window of the laser) by fitting a Gaussian profile. The result is shown in a form of a histogram in Fig. 4d. We found that the average width of the main electronic transition band for the measured single molecules is 35.5 nm, which is nearly 20% narrower (roughly 6 nm) than that of the ensemble absorption band (41.5 nm, indicated with vertical black line). The reduced width of the main electronic transition band for the single molecules clearly indicates that we are resolving the inhomogeneous broadening of the ensemble. The observed widths for individual molecules, however, might still be broadened due to spectral diffusion occurring during the acquisition time of the interferogram.

The observed variations in the excitation spectra are not because of random fluctuations in the experiment, or low signal-to-noise ratio as the variations between successively acquired excitation spectra on the same molecule are typically much smaller than differences in excitation spectra between different molecules.

### Simultaneous detection of excitation and emission spectra

**Figure 5:**
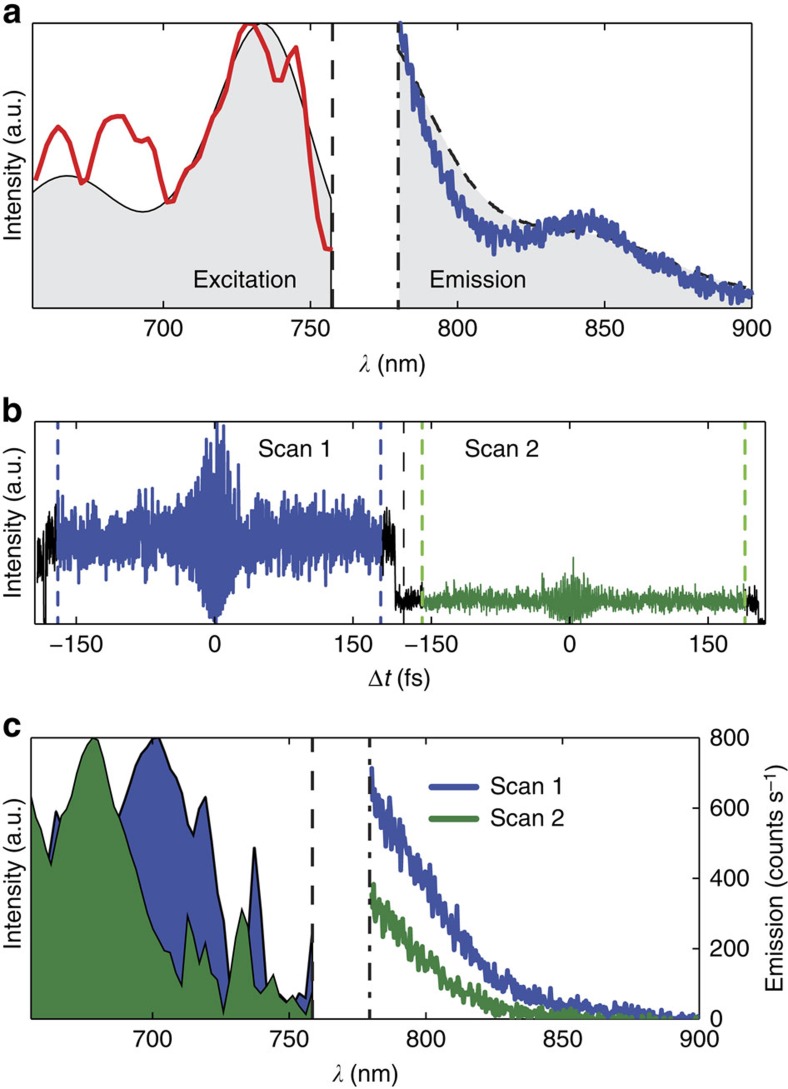
Simultaneous detection of emission and excitation spectra of individual molecules. (**a**) Exemplary excitation (red) and emission (blue) spectra measured simultaneously on the same molecule. For comparison we plot the QDI solution absorption and emission spectra (shaded grey) separated by the solution Stokes shift. (**b**) Two (blue and green) consecutive fluorescence interferogram scans measured on the same molecule. Interferogram sections marked with a black line are the parts ommited in the Fourier transformation analysis. (**c**) The corresponding excitation spectra alongside the simultaneously recorded emission spectra for each (blue and green) of the two scans. Vertical dashed and dash-dotted lines in **a** and **c** indicate cutoff wavelengths of the laser and long-pass filter, respectively.

Finally, we modified the experimental set-up to allow simultaneous detection of excitation and emission spectra of the same molecule at room temperature. To this end we placed a 50/50 beamsplitter in the fluorescence detection path and used half of the fluorescence signal for the detection of excitation spectra with the APD and the other half for the detection of emission spectra with the electron multiplying charge-coupled device (EMCCD) camera. In Fig. 5a we show the simultaneously recorded excitation (red) and emission (blue) spectrum of an individual QDI molecule. For comparison we also plot the QDI solution absorption and emission spectra (shaded grey). We spectrally offsetted both solution spectra to match the single-molecule spectra without changing the Stokes shift separation of the solution spectra. Both solution and single-molecule spectra match each other quite well. A part of the main peak of the emission spectrum is missing due to the cutoff wavelength of the long-pass filter used to filter out the laser light. For future experiments it might be advantageous to use a long-pass filter with a sharper cutoff slope and to move the cutoff point towards the middle of the Stokes separation.

QDI molecules are generally stable in their fluorescence emission; however, on a few occassions we observed jumps in the fluorescence intensity while recording fluorescence interferograms. On even fewer occassions such intensity fluctuations occurred towards the start or end of the recorded interferogram. A nice example of such discrete jump is shown in Fig. 5b. The investigated molecule clearly emits photons at distinct fluorescence levels. When analysing the two scans separately, the excitation spectra on the left side of Fig. 5c are obtained. They clearly have the main excitation peak at distinct spectral positions, separated by ∼20 nm. The corresponding, simultaneously acquired emission spectra are plotted alongside for both scans, showing similar shape but different intensity.

As this particular molecule absorbed and emitted more towards the blue side, we could not draw any direct conclusions on the spectral shift or position of the emission spectra, other than that we only detected the slope of the second emission band. However, we were able to compare the ratio in measured total fluorescence intensity to the ratio in excitation efficiency due to the spectral shift of the molecules' absorption spectrum. Taking the integrated product spectrum (which is the molecule's excitation spectrum times the laser spectrum), we found a ratio of 1/1.4 in excitation efficiency between the two scans. The ratio of fluorescence intensity between the two scans, however, is much larger and amounts to 1/4. It is thus clear that the emission spectrum must shift along with the excitation spectrum towards the blue, this way moving further out of the detection window (determined by the long-pass filter) and accounting for the lower detected fluorescence in the interferogram scans.

## Discussion

We have demonstrated that our broadband Fourier excitation approach effectively captures excitation spectra of individual molecules at room temperature. We, thus, put forward yet another way of detecting and investigating single molecules, right along with absorption, emission and scattering. The single-molecule excitation spectra allow us to resolve the intrinsic and environmentally induced inhomogeneities in the excited state potential of single molecules. These are inaccessible through emission spectroscopy (or Raman scattering techniques), which only probe the ground state potential. Furthermore, we have shown that the interplay between the excitation wavelength and detection spectral window may obscure the real extent of spectral inhomogeneity among single molecules, as indicated recently in the single-molecule experiment at cryogenic temperature^45^. One might fail to detect a significant fraction of the spectrally distributed molecules. This further corroborates the need of broadband excitation spectroscopy for the study of the single-molecule static and dynamic properties and their quantitative analysis of both emission and excitation spectroscopy.

The presented broadband excitation spectroscopy technique has the potential to contribute to the understanding of many dynamical processes such as intersystem crossing which typically affects the excitation spectrum but not the emission and absorption spectra. Consequently, we can probe the mechanisms and spectral dependence of intra- and intermolecular intersystem crossing, which so far has mainly been investigated at cryogenic conditions through statistical analysis of the blinking dynamics^46^,^47^,^48^,^49^,^50^ and through fluorescence-detected magnetic resonance spectroscopy^51^,^52^,^53^. Similarly, our approach offers new insights into single-molecule blinking dynamics. For single-molecule emission spectra acquired with a narrow excitation bandwidth, it is very challenging to differentiate between different blinking mechanisms like spectral jumps outside the excitation window and transitions to dark states. As we demonstrated, the broadband excitation spectroscopy approach allows us to follow the excitation spectrum in time and thus to verify correlations between blinking and spectral changes on the excitation side. We presented a proof-of-principal experiment, which demonstrates the feasibility of simultaneous acquisition of both the emission and excitation spectra and ambient conditions. We believe that this advancement in the single-molecule spectroscopy will allow for detailed studies on the interaction between individual molecules and their environment as well as for characterization of molecules in more complex systems. Finally, the technique will prove useful to follow slow-occurring chemical reactions in time through changes in the molecule's excitation spectrum both in solution and on the single-molecule level.

We note that our experimental approach is based on the concept of Fourier transform spectroscopy, however, with two important differences with respect to already established techniques. Firstly, commercially available FT spectrometers do not offer single-molecule sensitivity and do not allow for active control over excitation processes. As we use a coherent broadband light source we can manipulate the phase, time delay and chirp of the pulses. Transform limited pulses (in case of our set-up 12 fs) offer a time resolution that can be used to coherently probe femtosecond dynamics and (de)coherence of single molecules^54^,^55^. Using a laser with a sufficiently broad spectrum (exceeding the width of the absorption spectrum) it is possible to extract the dephasing time directly from the measured interferograms. Secondly, using two interfering excitation beams it should be possible to measure the excitation spectrum of non-fluorescent (single) molecules by detecting stimulated emission photons, a detection scheme which has already shown to reach nearly single-molecule sensitivity^56^. This would give access to the information normally obtained through fluorescence, but from non-fluorescent molecules.

On a technical note, the presented technique requires a broadband laser source and a simple (Mach–Zehnder) interferometer. However, it does not require any other modifications to a fluorescence detection scheme and thus is compatible with any confocal single-molecule detection set-up. It is worth noting that acquiring an excitation spectrum or an emission spectrum of a single molecule typically requires a similar number of photocounts and thus can be acquired in a comparable time. To obtain the presented excitation spectra of single molecules (with 4 nm resolution), a single interferogram was acquired for ∼120 s (see Methods). By reducing the sampling frequency and/or the data range (which decreases spectral resolution and spectral range) it is possible to record an interferogram within roughly 10 s without significantly affecting the shape of the measured excitation spectrum. The effect of reducing the resolution of the measured interferogram or its temporal range on the shape and quality of the excitation spectrum for molecule M4 (see Fig. 3), is shown in Supplementary Fig. 3. The acquisition time to obtain a reasonable fluorescence emission spectrum with the same excitation power is typically 2–10 s, depending on the brightness of the molecule. The few tens of seconds needed to measure an excitation spectrum of single molecules is comparable with the survival time of many biologically relevant fluorescing molecules. Therefore excitation spectra of molecules with low quantum effciency and photostability, like light harvesting complexes, is within reach.

## Methods

### Broadband excitation spectroscopy

The experimental set-up is schematically depicted in Fig. 2a. We used a broadband titanium-sapphire laser (Octavius-85M, Thorlabs) operating at 85 MHz and tuned to a central wavelength of 710 nm with a bandwidth of about 120 nm. The laser pulses were split into two parts in a Mach–Zehnder-type interferometer consisting of two identical 50/50 beamsplitters (Semrock) and a mechanical delay line (NRT 100/M, Thorlabs), which we used to control the path difference between the two arms of the interferometer. The interfering pulse pairs were propagated collinearly into an inverted microscope (Observer D1, Zeiss). A reference HeNe laser beam was propagated through the same interferometer, separated from the Ti:sapphire light using two band-pass filters (617±36, 632±11 nm, Semrock) and detected with a photodiode (PDA36A, Thorlabs). The derived reference interferogram was used to precisely determine the optical path difference between the interfering broadband pulse-pair. Before entering the microscope, the HeNe light was filtered out using a long-pass filter (635 nm LP, Semrock). In the microscope, the broadband Octavius pulses were reflected from a 50/50 beamsplitter and focused to a diffraction-limited spot on the sample with a high numerical aperture objective (1.3NA, × 100, Zeiss Fluar). The sample was placed on a piezo-controlled stage (Mad City Labs) allowing precise positioning of the molecules in the focal spot. The fluorescence from the sample was collected in reflection through the same objective and beamsplitter and sent either to a spectrometer equipped with an EMCCD camera for spectral detection (Newton, Andor) or to an avalanche photodiode (Perkin-Elmer) that allowed confocal optical imaging of the sample. The fluorescence was separated from the laser light using two long-pass filters (780 nm LP and 785 nm LP, Semrock). The laser interferograms were detected with a diffuser placed in front of an APD.

The experiment was performed as follows: first a confocal image of the sample was recorded. A molecule was placed in the focal spot, its position was optimized based on the intensity of the fluorescence signal and the interferogram scans were recorded until the molecule photobleached. Finally, the long-pass filter was replaced with the diffuser, the molecule was translated out of the focal spot and a series of reference laser interferograms was recorded.

### Detection efficiency

The laser power was set to roughly 1 μW for a single beam at the sample position, which corresponds to around 450 W cm^−2^. Considering transmission of optical elements in the detection path we were able to detect approximately 35% of the emitted fluorescence photons. For a typical QDI molecule this yielded 2–3 kcounts s^−1^. The delay line was scanned over 60 μm with an interpulse delay ranging from −200 to +200 fs. The delay line velocity was set to 0.5 μm s^−1^, which yielded a total single interferogram acquisition time of 120 s. The acquisition time was set to 10 ms, which including software data handling time, resulted in 12–13 measurement points per oscillation period of the broadband laser (corresponding to sampling rate of ∼5 × 10^15^ Hz or a measurement point every 0.15 fs). The resulting typical resolution in the frequency domain was 4.5 nm.

### Emission spectra

For the emission spectra, single molecules were excited by the HeNe laser (at 633 nm) using excitation intensity of 1–2 μW (450–900 W cm^−2^). The fluorescence was separated from the excitation light using a notch filter (633/25 nm) for the HeNe laser or a long-pass filter (690 nm cutoff) for the Ti:sapphire laser. Typically 20 emission spectra were recorded in a series with integration time of 11 s/spectrum. The gain of the charge-coupled device camera was set to 200. The emission spectrum of the QDI solution (10^−6^ M in 1% w/v toluene/PMMA) shown in Fig. 1b (shaded grey) was measured using HeNe excitation in combination with a 635 nm LP filter.

### Absorption spectra

The absorption spectrum of QDI (10^−6^ M) in toluene/PMMA (1% w/v) solution (Fig. 1b, shaded grey) was measured using a commercial spectrophotometer (NanoDrop 2000, Thermo Scientific). The absorption spectra as a function of evaporation (shown in Fig. 1b, also see Supplementary Fig. 4) were measured using the microscope's built-in halogen lamp. Light transmitted through the sample was collected through the objective and detected with the EMCCD camera. Typically 40 μl of solution (10^−5^ M) was placed on top of a microscope cover slip and a kinetic series of 8,000 transmitted light spectra was acquired, with integration time of 1 s per spectrum.

### Multicolour excitation

Confocal fluorescence images at different excitation wavelengths were recorded using a 5 nm broad excitation bands (in combination with a long-pass filter for detection): 570 nm (610 nm); 630 nm (648 nm); 680 nm (730 nm); 710 nm (740 nm); and 750 nm (778 nm); derived from an ultra-broadband laser (SuperK, NKT).

### Sample preparation

The QDI molecules were obtained from the Müllen group (Max Planck Institute for Polymer Research, Mainz, Germany). The samples were prepared by spin-coating a solution of QDI molecules at a roughly nM concentration in PMMA/toluene mixture (1% w/v). Approximately 50 μl of the solution was spin-coated on a #1 microscope cover slip for 60 s, at a spinning rate of 2,000 r.p.m. Before sample deposition, microscope cover slips were cleaned by leaving them in a piranha solution (1:2 ratio hydrogen peroxide to sulfuric acid) for about 30 min, then rinsing with deionized water and blow drying with nitrogen. We found this procedure to yield no or very little contamination on the cover slips.

## Additional information

**How to cite this article:** Piatkowski, L. *et al*. Broadband single molecule excitation spectroscopy. *Nat. Commun.* 7:10411 doi: 10.1038/ncomms10411 (2016).

## Supplementary Material

Supplementary Information Supplementary Figures 1-4 and Supplementary Note 1
