# Supplementary material for: Broadband single-molecule excitation spectroscopy
Source: Nat Commun. 2016 Jan 22;7:10411. doi: 10.1038/ncomms10411 (PMC4735816; doi:10.1038/ncomms10411)
Supplement: Supplementary Information — Supplementary Figures 1-4 and Supplementary Note 1 [file ncomms10411-s1.pdf]

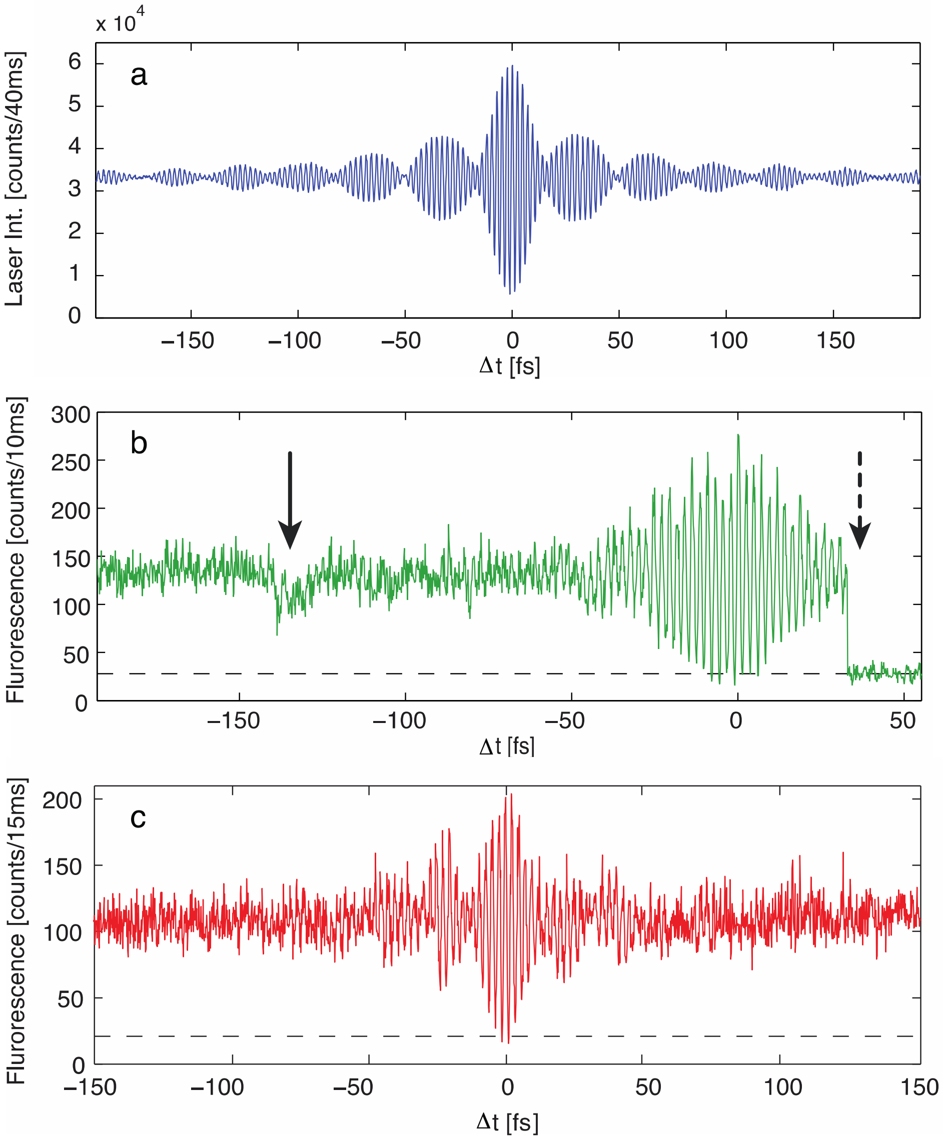

Supplementary Figure 1 Experimental interferometric excitation fluorescence scans. (a) An exemplary spectral interferogram of the laser pulse used in the experiment. **(b)** An example of a fluorescence interferogram of a molecule, which bleached during the first interference scan (marked by dashed, black arrow). Premature bleaching is often accompanied by fluorescence intensity fluctuations (marked with solid, black arrow). **(c)** A complete fluorescence interference scan measured on molecule M4 presented in the main text (see Figure 2b and Figure 3). The dashed lines in panel b and c indicate the background level.

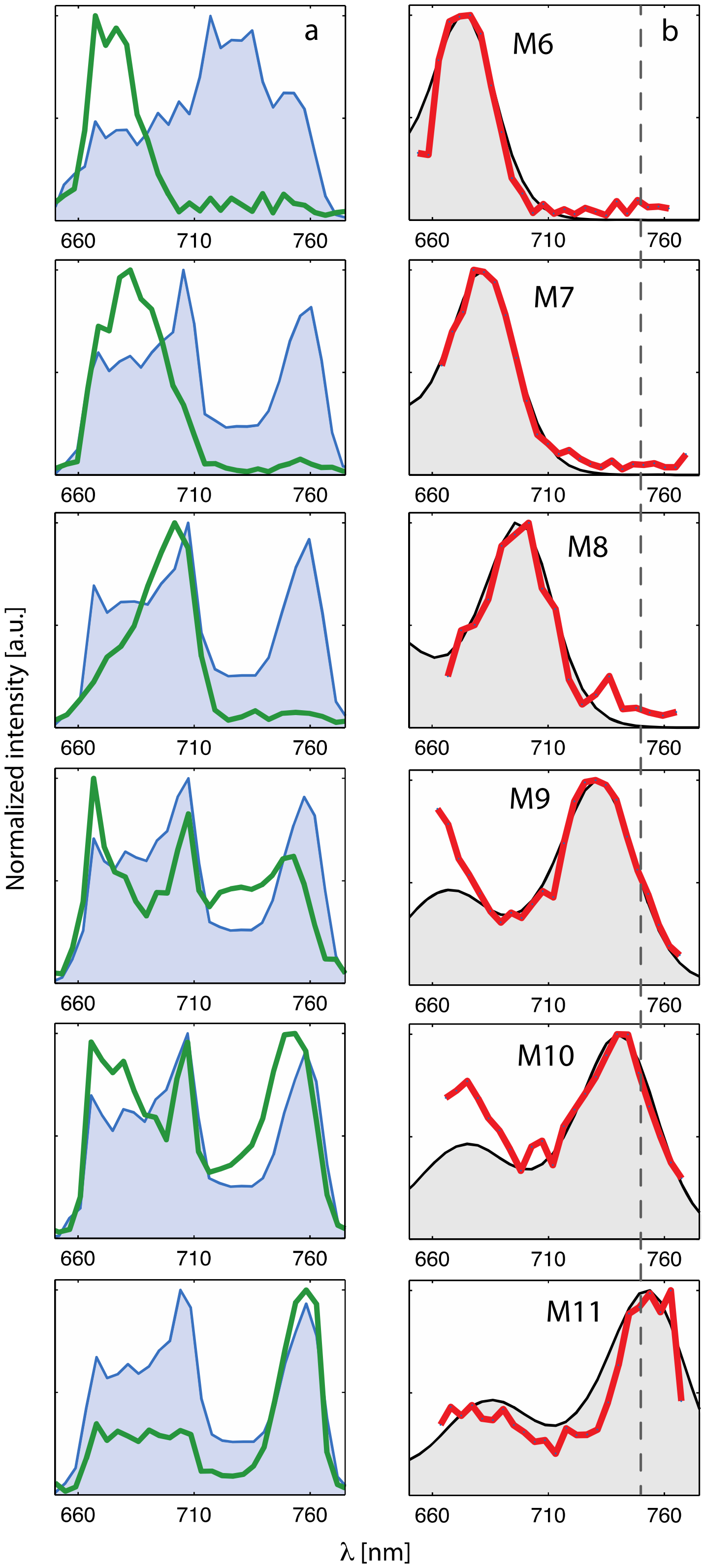

Supplementary Figure 2 Excitation spectra of single molecules. (a) An additional series of experimental product spectra (green) along with the used excitation laser spectra (shaded blue) and **(b)** the extracted excitation spectra of single QDI molecules (M6:M11, red lines). For comparison we show the spectrally blueshifted QDI solution absorption spectra (shaded, grey). The dashed line indicates the position of a maximum of the QDI ensemble absorption spectrum at 750 nm.

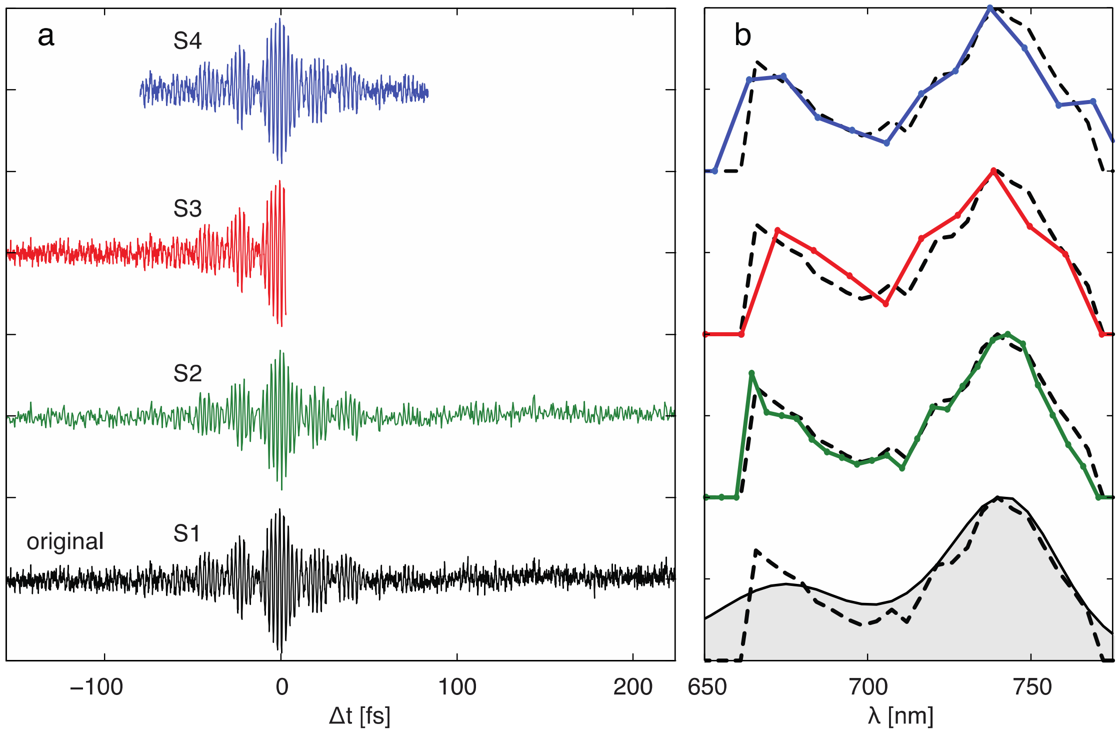

Supplementary Figure 3 The effect of the quality of the experimental interferograms on the shape and quality of the extracted SM excitation spectra. (a) A series of interferograms: the original one (S1), corresponding to molecule M4 (see Figure 3 in the main text) and its counterparts with altered length and resolution (S2, S3 and S4). S2 interferogram has 4 times lower resolution than the original S1. S3 and S4 have the same resolution as the original interferogram S1, but are 1/2 and 1/3 of the original range, respectively. **(b)** SM excitation spectra corresponding to the interferograms shown in panel **a**. Grey shaded is the QDI ensemble absorption spectrum. Marked with the dashed black line is the excitation spectrum corresponding to the original interferogram S1. The excitation spectra for interferograms with lower resolution and temporal length are shown with green, red and blue colors (S2->S3->S4). Clearly, no significant alteration of the shape of the excitation spectra is found if the resolution and/or temporal range of the interferograms are modified (to certain extent).

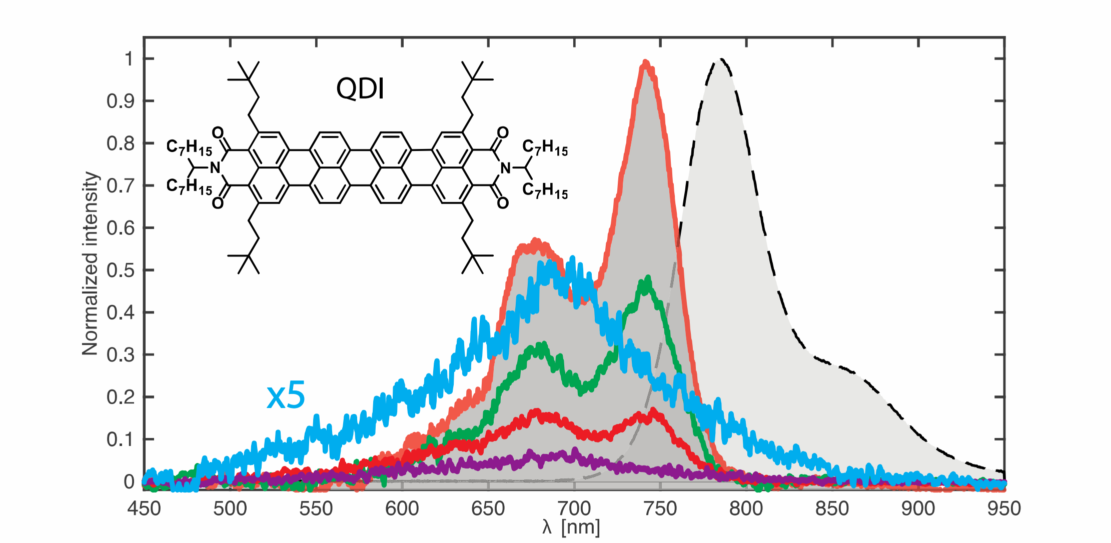

Supplementary Figure 4 Absorption spectra of QDI ensemble. The two grey, shaded curves represent absorption and emission (orange solid and black dashed, respectively) spectra of a solution of QDI molecules dissolved in PMMA/toluene mixture (1% PMMA w/v). The colored curves indicate the change of the absorption spectrum of the ensemble QDI solution upon solvent (toluene) evaporation (see Methods and Supplementary Note for experimental details). The orange curve indicates the initial solution spectrum directly upon deposition of the solution on the microscope cover slip. The intermediate absorption spectra are marked with the green, red and purple colors. The absorption spectrum of QDI molecules in solidified PMMA is shown in blue and scaled for clarity (x5). The inset shows the chemical structure of QDI.

Supplementary Note 1

In Supplementary Figure 4 we show the absorption (solid black line) and emission (dashed black line) spectrum of QDI in PMMA/toluene solution. The colored lines indicate the absorption spectra of QDI as a function of time (i.e. solvent evaporation and PMMA solidification). For experimental details, see Methods in the main text. Following deposition of QDI solution, we typically recorded 8000 spectra with an integration time of ~1 s/spectrum. Over the first few hundred spectra (~400) no changes other than a decrease in the intensity of the spectrum is observed (orange -> green spectrum). The reason for this is that the drop dries in a ring-like pattern (the 'coffee ring effect'). The middle part thus becomes thinner as the solution travels outwards. During the solidification of PMMA the absorption spectrum changes (see green, red and purple spectra), mostly loosing intensity on the higher wavelength side. Directly after that, the absorption spectrum of PMMA-encaged QDI molecules takes up a broad and featureless shape (blue spectrum, scaled x5 for clarity) spanning ~500 – 800 nm. We performed a principal component analysis on the recorded series of the absorption spectra and found only two significant components associated with the initial and final absorption spectrum. This final absorption spectrum seemingly lacks the vibrational progression that is well resolved in the solution absorption spectrum. The modification of the vibrational progression to such an extent is an unlikely explanation, as it would require changes in the vibrational energies in the same order of magnitude as the vibrational energy itself. It is

therefore more likely, as corroborated by our experiments, that the interactions between the QDI molecules and the surrounding PMMA affect the excitonic strength of the molecule and thus lead to a large spread of the spectral position. This in return leads to the washing out of the visibility of the vibrational progression of individual molecules. At the 10^{-5} M concentration we also cannot rule out completely that interfluorophore interactions affect the absorption spectrum. These, however, are absent in the single molecule level studies where much lower concentration is used (10^{-9} M).
